# Individual Differences in Scotopic Visual Acuity and Contrast Sensitivity: Genetic and Non-Genetic Influences

**DOI:** 10.1371/journal.pone.0148192

**Published:** 2016-02-17

**Authors:** Alex J. Bartholomew, Eleonora M. Lad, Dingcai Cao, Michael Bach, Elizabeth T. Cirulli

**Affiliations:** 1 Center for Applied Genomics and Precision Medicine, Duke University School of Medicine, Durham, North Carolina 27708, United States of America; 2 Department of Ophthalmology, Duke University, Durham, North Carolina 27710, United States of America; 3 Department of Ophthalmology and Visual Sciences, University of Illinois at Chicago, Chicago, Illinois 60612, United States of America; 4 Section Visual Function, Eye Center, Freiburg University, Freiburg, Germany; Eye Hospital, Charité, GERMANY

## Abstract

Despite the large amount of variation found in the night (scotopic) vision capabilities of healthy volunteers, little effort has been made to characterize this variation and factors, genetic and non-genetic, that influence it. In the largest population of healthy observers measured for scotopic visual acuity (VA) and contrast sensitivity (CS) to date, we quantified the effect of a range of variables on visual performance. We found that young volunteers with excellent photopic vision exhibit great variation in their scotopic VA and CS, and this variation is reliable from one testing session to the next. We additionally identified that factors such as Circadian preference, iris color, astigmatism, depression, sex and education have no significant impact on scotopic visual function. We confirmed previous work showing that the amount of time spent on the vision test influences performance and that laser eye surgery results in worse scotopic vision. We also showed a significant effect of intelligence and photopic visual performance on scotopic VA and CS, but all of these variables collectively explain <30% of the variation in scotopic vision. The wide variation seen in young healthy volunteers with excellent photopic vision, the high test-retest agreement, and the vast majority of the variation in scotopic vision remaining unexplained by obvious non-genetic factors suggests a strong genetic component. Our preliminary genome-wide association study (GWAS) of 106 participants ruled out any common genetic variants of very large effect and paves the way for future, larger genetic studies of scotopic vision.

## Introduction

The visual system operates over a remarkable range of lighting conditions through transduction by two classes of photoreceptor cells, rods and cones [1]. In photopic conditions, three types of cone photoreceptors with overlapping spectral sensitivities produce spatial acuity and color vision [2, 3]. In scotopic conditions, visual performance relies on rod photoreceptors, resulting in a reduction of spatial resolution of approximately 1200:1 in exchange for increased light detection sensitivity [4]. During mesopic vision, an intermediate illumination level between photopic and scotopic conditions, rod and cone pathways operate simultaneously to contribute to vision [5].

Visual acuity (VA) and contrast sensitivity (CS) are important measures of visual function, though the latter—while being a better predictor of traffic incidents [6]–is often neglected in clinical testing. VA describes spatial resolution under high contrast conditions, while CS describes the ability to distinguish small differences in luminance [7–9]. Visual performance can be differentially affected in differing luminance conditions, with performance in lower luminance conditions frequently being more sensitive to ocular dysfunction [8, 9]. Strong visual performance in mesopic or scotopic conditions tends to predict strong photopic vision, but the reverse does not necessarily hold true [7].

The majority of scotopic vision research to date has centered on diseased populations and treatment efficacy from refractive procedures such as laser eye surgery and intraocular lens transplantation [10]. In addition, it is known that scotopic vision can be adversely affected by a lack of essential nutrients such as Vitamin A and zinc deficiency [11, 12]. In healthy individuals, studies have preliminarily addressed the effects of age, pupil size, and astigmatism on differences in scotopic visual abilities [13–16]. However, no effort has yet been made to describe the individual differences in dark adaptation or scotopic visual function of healthy observers or to characterize the factors that influence these differences [9, 14, 17]. Differences between normal observers may result from rod density, differential convergence of rod signals, extent of activation of distinct pathways, functional differences in proteins such as rhodopsin, or other post-receptoral mechanisms [18, 19].

The contribution of a genetic component to healthy scotopic vision has yet to be explored, even though obvious heritable differences in photopic visual abilities exist [20, 21]. A host of genetic disorders, such as congenital stationary night blindness (CSNB) and retinitis pigmentosa (RP), are characterized by a compromise in rod function, resulting in night blindness [22–26]. The existence of Mendelian diseases affecting scotopic vision provides further evidence for a genetic influence on scotopic visual abilities [27, 28].

In this study, we first characterize normal scotopic VA and CS performance in a large population of healthy observers following 20 minutes of dark adaptation. We then define the contributions of a wide range of variables to scotopic performance. After controlling for confounding factors, we perform a preliminary genetic analysis using scotopic VA and CS as our outcomes. Our overall goal is to identify genetic variants with an influence on scotopic vision in healthy observers, leading to a better understanding of the complex molecular mechanisms involved in scotopic vision.

## Materials and Methods

### Participants and Ethics

The Duke University Institutional Review Board approved all procedures, and participants provided written, informed consent (IRB #: Pro00006828).

A total of 734 participants attempted the visual tasks as part of a larger battery in the Duke Genetics of Cognition and Other Normal Variation study [29–31]. Due to the complex protocol for night vision testing, complete and useable visual data was available for 664 individuals due to participant, examiner, or device error. Further exclusionary criteria required to define a healthy population of normal observers resulted in a final sample size of 501 (criteria described below). A comprehensive description of these 501 participants can be seen in Table 1.

**Table 1:** Participant Demographics.

Variable	Mean (SD) or Count (%)
Age in years	22.8 (4.3)
Ancestry	
European	237 (47.3%)
African	60 (12.0%)
East Asian	72 (14.4%)
Hispanic	47 (9.4%)
Other	22 (4.4%)
Sex	
Female	321 (64.1%)
Education	
Years of education	15.1 (1.9)
Current student	377 (75.3%)
Astigmatism	91 (18.2%)
BDI score > 14	23 (5.1%)
Color vision deficiency	7 (1.4%)
Iris color	
Blue-gray	59 (16.6%)
Green-hazel	60 (16.9%)
Brown-black	236 (66.5%)
Laser eye surgery	12 (2.4%)
CIRENS	0.57 (1.21)
Fatigue	2.42 (0.87)

Standard deviation (SD), Beck Depression Inventory (BDI), Circadian Energy Scale (CIRENS). Note that only 451 the 501 included participants had BDI scores, and only 355 had iris color responses.

### Questionnaire

Prior to psychometric and visual testing, participants completed an extensive survey that queried demographics, medical history, and several standardized scales. To identify new variables associated with scotopic vision, we investigated multiple measures for association with this phenotype. Many of these variables, such as years of education and ethnicity, have never before been compared to the scotopic visual performance of a person.

### Depression

A total of 451 participants completed the Beck Depression Inventory-II (BDI) [32]. Participants scoring 14 or higher were categorized as depressed.

### Circadian rhythms

The Circadian Energy Scale (CIRENS) is a two-question chronotype measure based on self-report energy levels throughout the day: once at night and once in the morning. Energy levels are described on a Likert scale: [very low (1), low (2), moderate (3), high (4), or very high (5)]. The difference between the evening score and morning score determines the overall chronotype score, ranging from -4 (most marked morning preference) to +4 (most marked evening preference) [33]. Previous studies have shown changes in night vision phenotypes during different times of day and parts of the Circadian cycle [34, 35].

### Fatigue

Prior to visual tasks, participants indicated their current level of tiredness on a Likert scale: [energetic (0) to very tired (4)].

### Ocular information

Participants self-reported eye health and characteristics. For iris color, participants were divided into one of three groups: a baseline group of participants indicating brown or black irises, a second group with green or hazel irises, and a third group with blue or gray irises. Previous studies have investigated the impact of iris color on certain aspects of ocular function but have not investigated whether it is associated with scotopic vision [36–38]. Participants also self-reported whether they were previously diagnosed with astigmatism in the eye tested or had a color vision deficiency, and provided a history of ocular disease or interventional procedures. No further assessment of these traits was performed beyond the self-report.

### Cognitive test

Participants completed a cognitive battery assessing diverse areas of cognition as previously described [29]. Principal component analysis was performed on the eleven individual test scores to determine an overall measure of performance [29]. The first principal component explained 37.3% of the total variance in test scores and received approximately equal loadings from all tests. It was therefore taken as a measure of overall cognitive performance on the battery and can be considered a proxy for general intelligence.

### Freiburg Visual Acuity and Contrast Test (FrACT)

VA and CS thresholds were assessed using the Freiburg Visual Acuity and Contrast Test (FrACT; Version 3.7.4.c) [39]. FrACT employs a best parameter estimation by sequential testing (best PEST) algorithm to adapt optotypes in real time to the user. FrACT displays Landolt Cs over a large range of VA and CS in an eight alternative forced choice task [40]. FrACT has been utilized in over 400 publications and is available free of charge at michaelbach.de/fract.html. To assess VA, the size of a high-contrast Landolt-C is varied. The size of the gap at threshold, measured in minutes of arc, is taken as the minimum angle of resolution (MAR); its logarithm (“logMAR”) is a standard measure of visual acuity [41]. To assess CS, the contrast of a large (3.3° diameter) Landolt-C is varied, while its size remains constant. The contrast at threshold is initially expressed as fractional Weber contrast C_W_ (C_W_ = luminance difference divided by the surround luminance); its value is typically around 0.01 for photopic vision and 20 times higher for scotopic vision. [For isolated optotypes on a large background the Weber contrast is more relevant than the Michelson contrast.] This contrast threshold is converted to log contrast sensitivity (logCS_Weber_ = log(1/C_W_)).

Participants first completed both the FrACT VA and CS tasks twice in photopic conditions. The tests were given twice to allow assessment of test-retest agreement and to improve our ability to identify outliers. Participants then dark adapted for 20 minutes and then completed the FrACT VA and CS an additional two times. Participants were required to finish both repeats in five minutes to allow for a standard start time for the next visual task and to standardize the dark adaptation length for all participants; if they did not complete both, then only one measure was used.

The same computer was used for both photopic and scotopic tests. The results were obtained with a 17” monitor at a distance of 1.54 m. The diameter of the Landolt C was set to 200 minutes of arc (arcmin) at a constant optotype contrast of 100%. For high reliability, a total of 24 trials for each task were used [40]. The monitor luminance in photopic conditions was 90 cd/m², and the ambient lighting was 300 lux. Luminance was measured using the i1Display 2 (X-Rite, Grand Rapids, MI). For scotopic conditions, the ambient lights were extinguished, and great care was taken to eliminate all potential sources of light from the dark room. Neutral density filters (Paul C. Buff, Inc., Nashville, US) were placed over the computer monitor, reducing the monitor luminance to 0.00092 cd/m².

Results for all visual tasks are monocular and reflect performance in the participant’s right eye (n = 490) unless a participant self-reported worse vision compared to the left eye. The amount of time spent per task was recorded for FrACT in ms and log-transformed for analysis; referred to as ‘task duration’. We did not use a fixation task but attempted to minimize variation in testing strategies by informing participants to not continuously fixate on the stimulus. Auditory feedback was provided to indicate correct or incorrect.

#### Absolute detection threshold

Following FrACT completion that accounted for 25 minutes of dark adaptation, 248 participants completed the absolute detection threshold measurement using a LED-based dark adaptometer built in Dr. Cao’s laboratory. The test size was 4° in visual angle and included a 10° temporal eccentric fixation. The stimuli were 100ms pulse square waves with a dark background (i. e. 0 cd/m^2^); output was digitally controlled by an M-Audio PCI soundcard [42]. Two independent thresholds were generated using a two-yes/one-no staircase procedure that varied the peak luminance of the square wave until the threshold was determined. The smaller of the two thresholds served as the dark adaptation (DA) phenotype.

In addition to the exclusionary criteria (described below) to define a healthy population, 14 participants were also excluded from DA analyses because their threshold was more than 3 SD above the group mean, indicating greatly impaired scotopic vision. Our final DA sample size was 234 participants.

### Repeat Sessions

**Fig 1:**
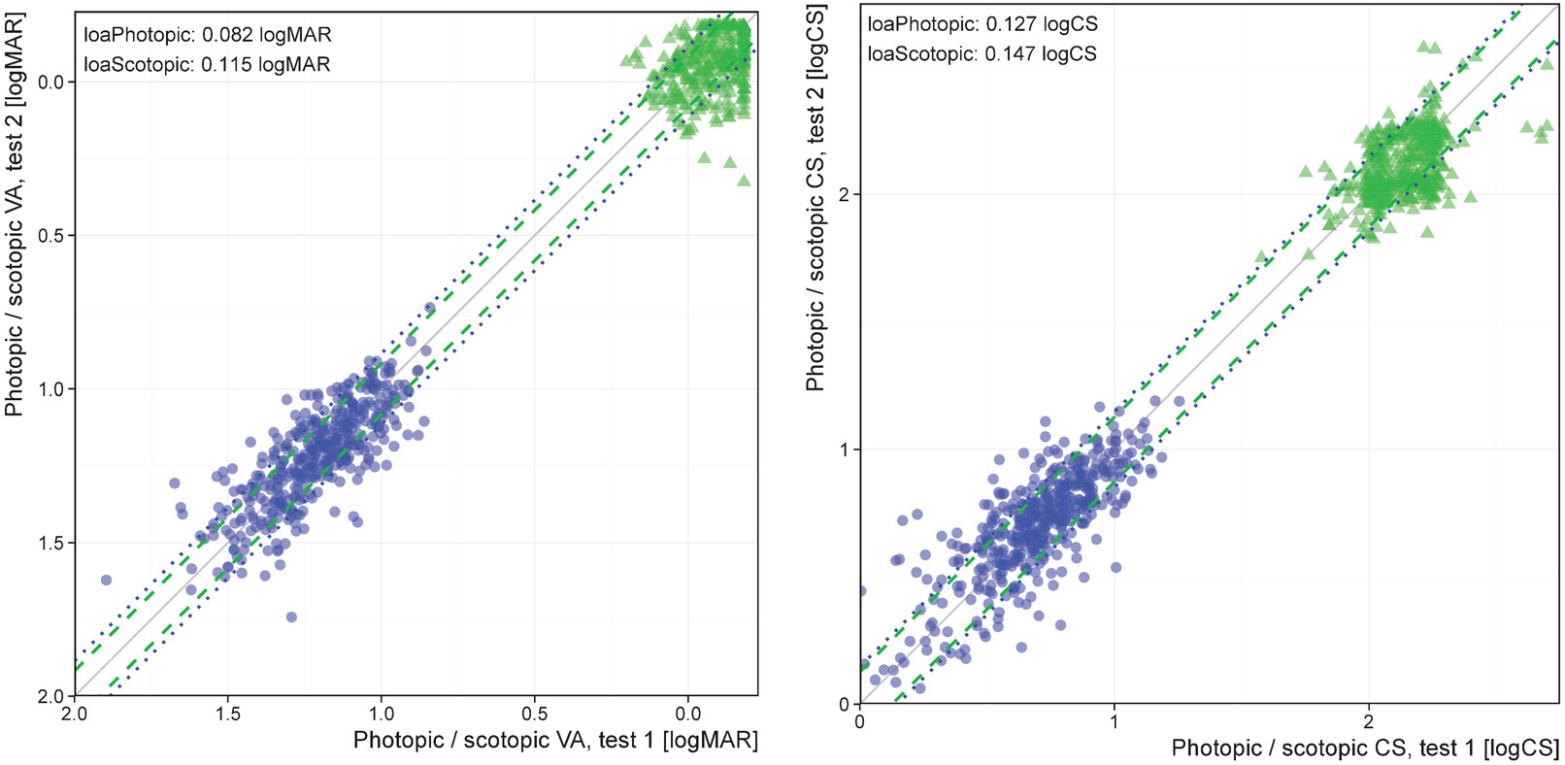
Test-retest assessment. Four data sets are depicted: Visual acuity (left panel) and contrast sensitivity (right panel) at photopic luminance (green triangles, near top left and at scotopic luminance (blue discs, near bottom left). Result of the first test on the abscissa, second test on the ordinate. Grey 45°-line is the identity line, next to it the ± limits of agreement (photopic, dashed; scotopic, dotted). Visual acuity in logMAR units have an inverted scale, and contrast sensitivity is in logCS_Weber_ units, meaning that better performance corresponds to the top right for both graphs. As expected, photopic measures of VA or CS are markedly better than scotopic ones. The 95% limits of agreement are remarkably similar. All in all, there is no marked deviation from a normal distribution, and the reliability is good for the range measured.

To evaluate the reliability of our tasks, 40 participants completed both the FrACT and DA tasks at two separate testing sessions, a mean of 85 days apart (SD = 18). We were also able to evaluate test-retest agreement within the same testing session as most participants completed two photopic and scotopic scores during the initial testing session (Fig 1).

### Data Analyses

#### Phenotypes

The primary outcomes were FrACT phenotypes, which were generated based on the average of two independent thresholds for photopic conditions and for scotopic conditions after twenty minutes of dark adaptation. When only one photopic or scotopic score was recorded (n = 102), this measure was used as the phenotype.

For the test-retest agreement, we calculated the 95% limits of agreement [43].

#### Exclusionary criteria

Useable data were generated for 664 participants who were judged by our psychometricians to fully comprehend the task and have no technological or methodological barriers to performance. To define a healthy population of observers, those with photopic VA worse than 20/25 (corresponding to ≥ 0.1 logMAR), who were older than age 40, who did not recognize any optotypes after 20 minutes of dark adaption, or who were more than 3 SD beyond the mean scotopic VA performance were excluded from all analyses.

In addition, those with previous laser eye surgery were excluded from genetic analyses because this procedure had significant impact on scotopic performance in our task, concordant with the pertinent literature and potentially due to dry eyes [44–46].

#### Statistical analyses

All non-genetic statistical analyses were performed using STATA 13.1 [47]. Stepwise forward linear regression analyses with a cutoff for inclusion of *p* < 0.01 were performed for the VA and CS scotopic phenotypes, with the phenotype as the outcome and variables listed in Table 2 as covariates. Scotopic VA population passed Shapiro-Wilks (*p* > 0.001) for normal distribution. The scotopic CS population did not pass Shapiro-Wilks (*p* < 0.001) due to a tail of low performers. Scores were normally distributed when restricting to those who performed no more than 2 SD below the mean (n = 25). This restriction had no significant impact on the predicted stepwise model, so we retained these 25 individuals in the analysis.

**Table 2:** Associations with scotopic VA and CS.

Variable	Scotopic Visual Acuity						Scotopic Contrast Sensitivity					
	Univariate analyses			Multivariate analysis with covariates from stepwise model			Univariate analyses			Multivariate analysis with covariates from stepwise model		
	*p*	*β*	R^2^	*p*	*β*	R^2^	*p*	*β*	R^2^	*p*	*β*	R^2^
Task length (VA or CS)	**< 0.001**	-0.402	0.270	**	**	**	**<0.001**	0.508	0.265	**	**	**
Photopic performance (VA or CS)	**< 0.001**	0.400	0.041	**	**	**	**<0.001**	0.284	0.025	**	**	**
Laser eye surgery	**0.002**	0.141	0.019	**	**	**	**0.004**	-0.167	0.017	**	**	**
Intelligence	0.020	-0.008	0.011	**	**	**	**0.004**	0.013	0.017	**	**	**
Age	NS			NS			NS			NS		
Ancestry			0.035	NS					0.044	NS		
African	0.111	0.036					0.033	-0.060				
East Asian	0.001	0.070					<0.001	-0.100				
South Asian	0.021	0.056					0.002	-0.095				
Hispanic	0.063	0.064					0.052	-0.084				
Other	0.746	-0.009					0.395	-0.023				
Education			0.010	NS			NS			NS		
Years of education	0.492	0.003										
Current student	0.027	0.038										
Male	NS			NS			NS			0.050	0.030	0.335
Astigmatism	NS			NS			NS			NS		
Eye color#	NS			NS			NS			NS		
Blue-gray												
Green-hazel												
Depression#	NS			NS			NS			NS		
CIRENS	NS			NS			NS			NS		
Time of day	NS			NS			NS			NS		
Bin 1 (9:00–11:00am)												
Bin 2 (11:00am– 1:00pm)												
Bin 3 (1:00pm– 3:00pm)												
Fatigue	NS			NS			NS			NS		

Bolded *p* values are < 0.01. NS indicates p > 0.05.

^#^ Not included in multivariate stepwise regressions because missing from some participants as detailed in the methods.

** Included as a covariate in all multivariate regressions due to significant association in stepwise regression

Two variables, BDI and iris color, were unavailable for some participants and were subsequently excluded from the stepwise analyses to maintain the largest sample size possible. A total of 50 individuals were missing BDI score, and 146 individuals did not have iris color responses. These variables were not found to be significantly associated with the phenotypes in univariate analyses.

Variables that were significant in the stepwise model were used as covariates in subsequent genetic analyses and in the multivariate analyses results presented in Table 2.

#### Genetic analyses

A genome-wide association study (GWAS) was performed on 106 participants who had Illumina Humanexome chip data available. Of these, most were also genotyped with the Infinium HumanCore GWAS chip (n = 93), and others were genotyped with either the Human610-Quad BeadChip (n = 6) or HumanHap550 (n = 5). Two of these 106 samples did not have GWAS genotypes. Variants from any of these chips were included in the analysis provided they passed QC and met the below inclusion criteria.

Our single variant analysis restricted to variants genotyped in at least 50% of these participants with a minor allele frequency (MAF) of at least 0.01 A linear regression was used in PLINK [48] for scotopic VA and CS performance (n = 106). Two EIGENSTRAT axes [49] and significant variables from the stepwise models were used as covariates. A total of 273,230 variants were analyzed in this GWAS [16]. Correction for multiple tests therefore required a p-value of 1.8x10^-7^ to reach significance. We also performed a focused analysis on 94 candidate genes annotated as being involved in the phototransduction and retinol metabolism pathways [50, 51] as well as 55 candidate genes implicated in Mendelian diseases causing night vision defects (http://omim.org/).

To assess the effects of the low frequency variants genotyped with the exome chip, we used a gene-based collapsing analysis as previously described [52]. Briefly, we summarized for each participant whether there existed a ‘qualifying’ variant in each gene, where qualifying was defined as an exonic variant with MAF < 0.01. Linear regression analysis was then performed with two EIGENSTRAT axes, the task duration, and the photopic score, and the first principal component of the cognitive battery as covariates. This allows the identification of genes where qualifying variants are enriched in individuals toward one extreme or the other of each trait. Of the candidate genes described above, 32 had low frequency coding variants included in this gene-based collapsing analysis.

Power calculations were performed using GWASpower/QT [53] (available at http://igm.cumc.columbia.edu).

## Results

### FrACT test-retest agreement

Test-retest agreement of FrACT photopic and scotopic VA and CS were evaluated from 399 participants who completed the tasks twice at the initial testing session (Fig 1) and an additional 40 participants who repeated the tasks at a later session (S1 Fig). As expected, photopic measures of VA or CS were superior to scotopic ones. The 95% limits of agreement for all conditions at the first testing session are remarkably similar. For VA, photopic: 0.082 logMAR, scotopic: 0.115 logMAR, thus corresponding to ±1 line on an acuity chart. For CS, photopic: 0.127 logCS_Weber_, scotopic: 0.147 logCS_Weber_. Performance between the two testing sessions was also highly consistent: the 95% limits of agreement are 0.089 for photopic, 0.124 logMAR for scotopic VA, 0.165 for photopic, and 0.242 logCS for scotopic CS.

### Photopic FrACT VA and CS performance

**Fig 2:**
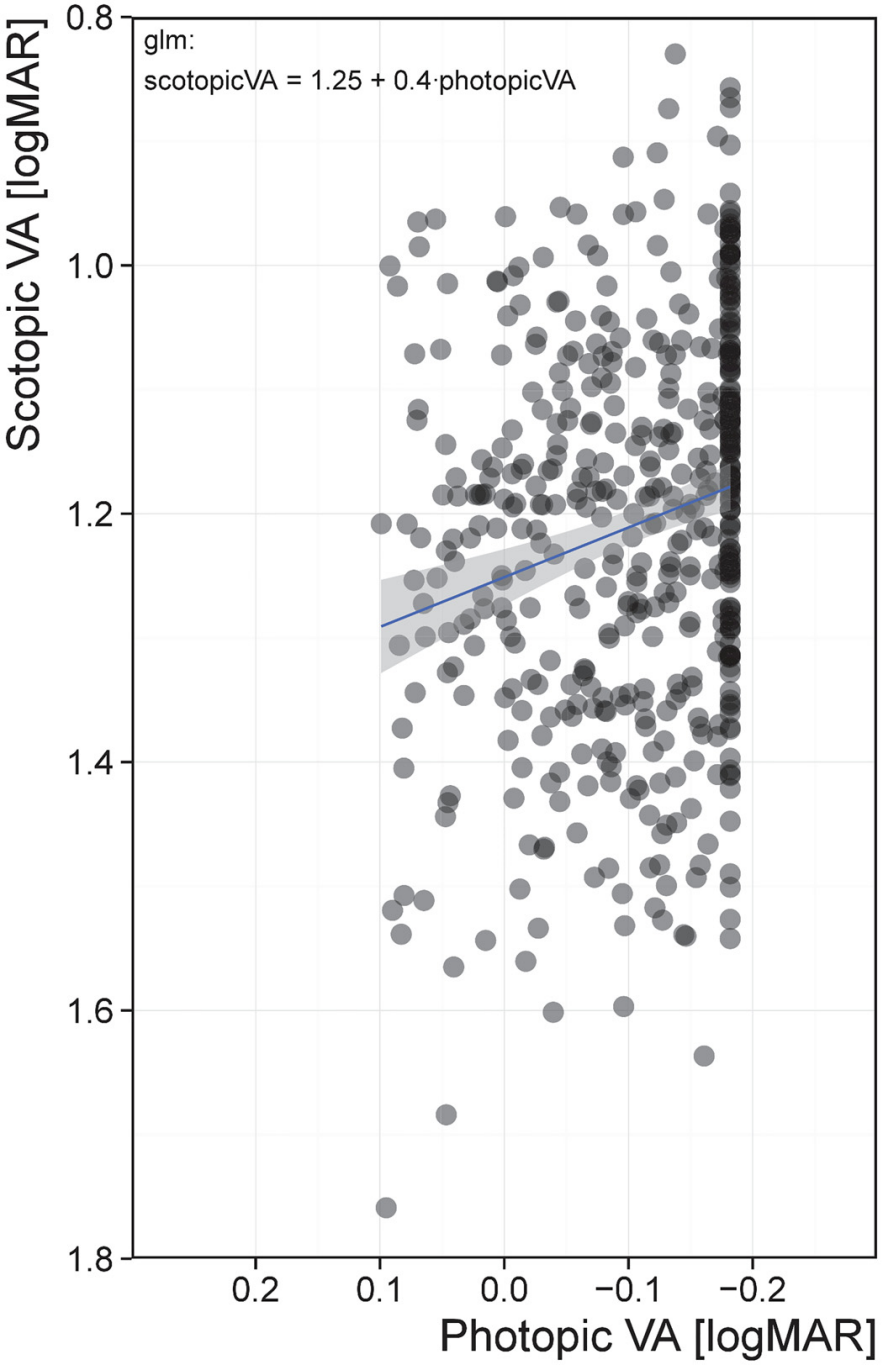
Correlation between scotopic VA and photopic VA. Visual acuity in logMAR units have an inverted scale, meaning that better performance is shown here with a higher score. Photopic VA explained 4.1% of the variance in scotopic VA.

**Fig 3:**
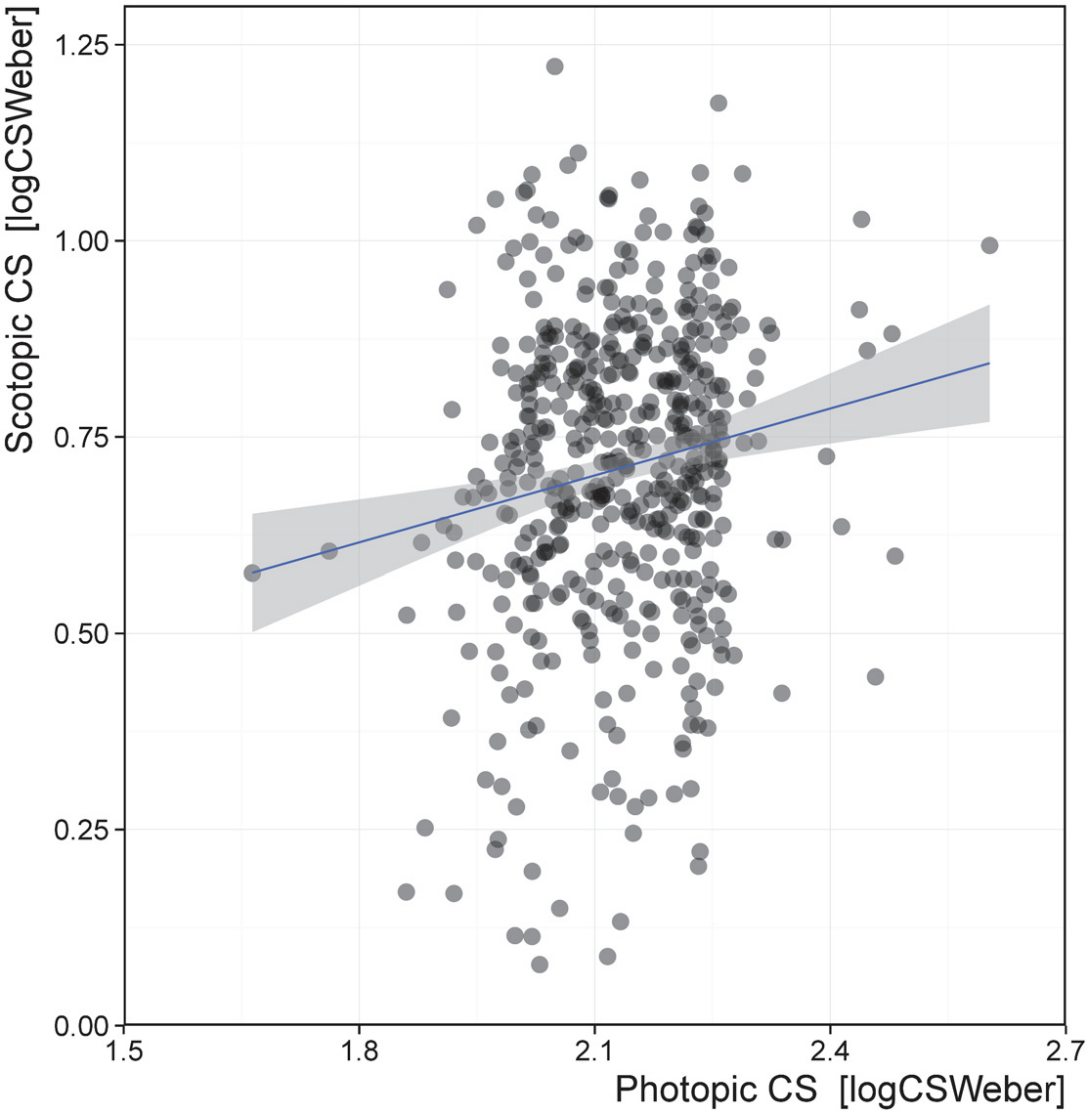
Correlation between scotopic CS and photopic CS. Contrast sensitivity is in logCS_Weber_ units, meaning that better performance is a higher score. Photopic CS explained 2.5% of the variance in scotopic CS.

Photopic VA scores exhibited a ceiling effect at -0.182 logMAR (mean = -0.105, SD = 0.080) with 30.7% of the final population arriving at this threshold. This ceiling effect was due to the pixel resolution of the computer monitor at the testing distance used. VA performance in photopic conditions was significantly correlated with VA performance in scotopic conditions (*p* < 0.001) but explained only 4.1% of the variance (Fig 2). Photopic CS scores ranged from 1.66 to 2.60 logCS_Weber_ (mean = 2.13, SD = 0.110); performance was significantly correlated with scotopic CS (p < 0.001) but explained only 2.5% of the variance (Fig 3).

Individual performance on photopic VA and CS was significantly correlated (*p* < 0.001), and scores on one explain 9.8% of the variance in the other.

### Scotopic FrACT performance and absolute detection threshold

**Fig 4:**
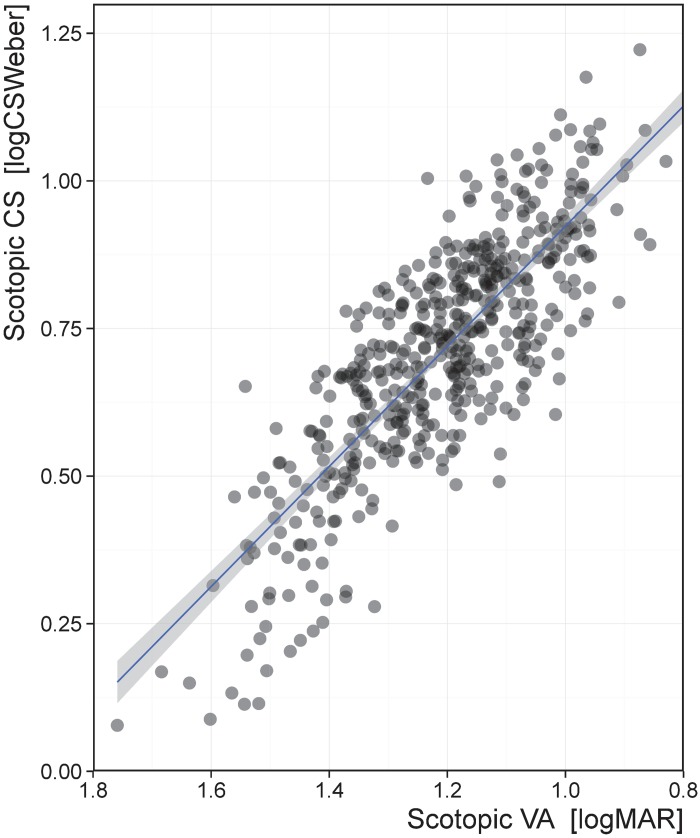
Correlation between scotopic VA and scotopic CS. Visual acuity in logMAR units have an inverted scale, and contrast sensitivity is in logCS_Weber_ units, meaning that better performance corresponds to the top right. Scotopic VA explained 67.1% of the variance in scotopic CS.

Scotopic VA performance was significantly correlated with scotopic CS performance (*p* < 0.001; Fig 4) and explained 67.1% of the variance. Scotopic VA (mean = 1.21, SD = 0.159) ranged from 0.79 to 1.76 logMAR and scotopic CS (mean = 0.711, SD = 0.197) ranged from 0.08 to 1.22 logCS_Weber_. We additionally found that scotopic VA and CS performance was significantly correlated with the absolute detection threshold on the dark adaptometer (VA: r = 0.31, *p* < 0.001; CS: r = -0.30, *p* < 0.001).

### Multivariate stepwise regressions: scotopic FrACT VA and CS

Stepwise regression analyses assessed the contribution of a number of demographic variables, eye characteristics, photopic visual performance, task duration, time of day, and intelligence to scotopic VA and CS (Table 2). Variables meeting inclusion criteria for the VA and CS models explained a total of 32.7% and 32.9% of the variance, respectively. For both models, the strongest predictor of scotopic performance was task duration (*p* < 0.0001), accounting for a total of 27.0% of the variance in VA and 26.5% in CS performance. Three additional variables passed inclusion criteria for both models (*p* VA; *p* CS): photopic VA or CS (<0.0001; <0.0001), intelligence (0.0014; <0.0001), and previous laser eye surgery (0.0009; 0.0033).

### Genetic associations

After correcting for multiple tests, we identified no variants or genes with statistically significant associations with these traits (*p* < 1.8x10^-7^). Our genome-wide association study had 80% power to identify a common variant explaining at least 25% of the variation in this trait, and our gene-based collapsing analysis of low-frequency coding variants had 80% power to identify associations explaining at least 22% of the variation. When restricting to 139 candidate genes annotated as being involved in the phototransduction and retinol metabolism pathways or implicated in Mendelian diseases causing night vision defects, we still found no significantly associated variants. However, we did identify a trend for participants with rare coding variants in *RP1* to exhibit worse scotopic CS (corrected p = 0.018; VA corrected p not significant). This association was driven by three nonsynonymous variants—rs137887415, rs16920621, and rs142318038—each of which had one heterozygous carrier in our dataset.

## Discussion

This study characterized the performance of 504 healthy observers on scotopic VA and CS and analyzed numerous factors with the potential to affect performance, most of which had not previously been assessed with regard to this phenotype. Focusing on young individuals with excellent photopic vision, we found a wide variation in performance (Fig 1) that is largely unexplained by a range of factors such as Circadian preference, photopic visual performance, intelligence, or eye characteristics. Combined with high test-retest agreement and the existence of diseases uniquely targeting rod systems, our findings argue for a strong genetic component of healthy variation in night vision that demands exploration.

**Fig 5:**
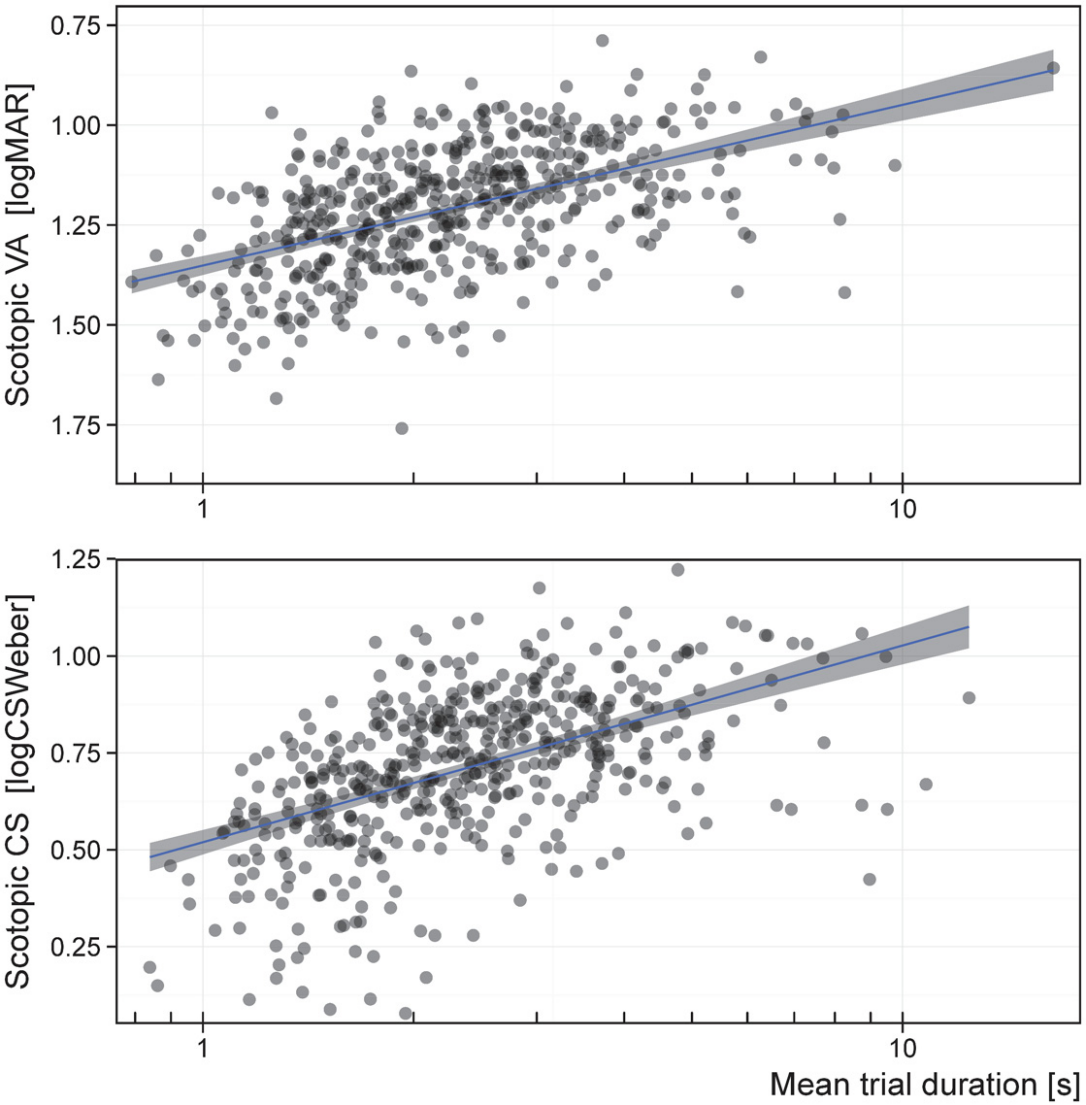
Correlation between scotopic VA, scotopic CS, and task duration. Visual acuity in logMAR units have an inverted scale, and contrast sensitivity is in logCS_Weber_ units, meaning that better performance corresponds to higher scores. Task duration is shown on a log scale and explained 27% of the variance in scotopic VA and CS.

It is clear that the amount of time spent observing the optotype plays a significant role in scotopic conditions in both the VA and CS test. The amount of time that the participant chose to spend observing the optotype before deciding how to respond is essentially their reaction time, and here we represent this concept as overall task duration. Note that the number of trials in the VA and CS tests used here was fixed, as was the dark adaptation time for each participant, so the relationship between task duration and performance is not a product of differences in the number of trials or the dark adaptation length. While the duration of optotype presentation has been previously documented to impact photopic performance, ours is the first study to show a strong effect under scotopic conditions [54]. In fact, our study shows a much stronger effect of task duration on scotopic performance than on photopic performance (Fig 5 and S2 Fig). In addition, we find intelligence to be a predictor of scotopic performance, which may reflect an increased ability to navigate this testing paradigm or reflect different approaches or level of interest in the testing according to intelligence. Intelligence was also positively correlated with task duration, indicating that higher intelligence may have led to improved testing strategies. Despite the significance of these variables to performance, they collectively only explain < 30% of the variation in scotopic VA and CS, leaving the vast majority of the variation in scotopic visual performance unexplained.

Our results confirm that individuals who have undergone laser eye surgery perform significantly worse on both scotopic VA and CS in addition to previously reported night vision issues in such patients [44, 45, 55]. In addition, photopic performance was significantly correlated with scotopic performance, but only explained < 5% of the variance. In a previous study of 47 normal observers, CS performance under mesopic and photopic conditions was also significantly correlated, with 27% of the variance in common [7]. This work documented a notable range of mesopic CS performance that was independent of photopic CS performance. We confirm and extend this finding into scotopic conditions for CS, and we demonstrate the same marked dissociation between scotopic and photopic VA.

In addition, the results demonstrate no effect of astigmatism, iris color, Circadian preference, depression status, time of day, fatigue or color vision deficiency on scotopic performance. We were underpowered to assess the effects of color vision deficiency as our study only contained 7 individuals; the mean performance of these participants was in line with that of the remainder of the population for both VA and CS tasks. This finding is concordant with recent work demonstrating no expected effect of color vision deficiency [56], but stands in opposition to older research demonstrating lower light perception thresholds in these individuals [57]. We do find worse scotopic VA and CS performance in those of Asian ancestry when performing univariate regression analyses, but these associations were no longer statistically significant after accounting for laser eye surgery, task duration, intelligence, and photopic performance.

The preliminary genetic analyses carried out utilized low frequency coding variants as well as common GWAS variants. We performed both single variant analyses and gene-based collapsing analyses that combined the effects of all rare coding variants in each gene. Our focused analysis of candidate genes identified a trend for three participants with rare coding variants in *RP1* to exhibit worse scotopic CS. Loss of function mutations in this gene are known to cause autosomal dominant retinitis pigmentosa, a retinal disease with night blindness and progressive retinal degeneration [58, 59]. The variants in our study are not loss of function mutations: they are rare nonsynonymous variants, most of which are predicted by Polyphen2 to be benign [60]. This preliminary observation warrants follow-up in additional samples to determine whether variation in this gene impacts the normal range of human scotopic vision. Our genome-wide analysis was powered to identify associations explaining at least 25% of the variation in scotopic vision, and we did not identify any variants or genes with such a large effect. This result is not unexpected given the complexity of night vision, but it does lay the groundwork for the types of sample sizes and genetic data that will be required for future studies of this trait.

While our study was large in size, it may have been limited by self-report responses given by participants. We do not have access to the medical records of our participants, and they did not undergo full ophthalmologic exams to determine the presence of any organic eye disease beyond those that are self-reported. In future studies, a full ophthalmic assessment of the participants with indications of organic eye disease or poor performance would be helpful for confirming and extending our findings.

Our study demonstrates the wide variation that is seen between healthy volunteers in their scotopic visual performance, even after controlling for age and photopic vision. We confirm that this inter-individual variation is stable and largely uninfluenced by known factors, which strongly supports there being a genetic component to these scotopic visual traits. Future studies may expand on these findings through a comprehensive, large-scale characterization of scotopic vision in healthy observers to continue assessing the impact of genetics on visual acuity and scotopic visual performance.

## Supporting Information

S1 Fig Test-retest assessment over different days. The tests presented here were taken a mean of 87 days apart (SD = 21). Four data sets are depicted: Visual acuity (left panel) and contrast sensitivity (right panel) at photopic luminance (green triangles, near top left and at scotopic luminance (blue discs, near bottom left). Result of the first test on the abscissa, second test on the ordinate. Grey 45°-line is the identity line, next to it the ± limits of agreement (photopic, dashed; scotopic, dotted). Visual acuity in logMAR units have an inverted scale, and contrast sensitivity is in logCS_Weber_ units, meaning that better performance corresponds to the top right for both graphs.
(PDF)

S2 Fig Correlation between photopic VA, photopic CS, and task duration. Visual acuity in logMAR units have an inverted scale, and contrast sensitivity is in logCS_Weber_ units, meaning that better performance corresponds to higher scores. Task duration is shown on a log scale and explained <0.1% of the variance in scotopic VA and 3.3% of the variance in scotopic CS.
(PDF)

S1 File Phenotypes and Covariates. Phenotypic and covariate information for the samples included in this study.
(XLSX)

S2 File Collapsing Analyses. Results from the gene-based collapsing analyses included in this study.
(XLSX)

S3 File GWAS Analyses. Results from the GWAS analyses included in this study.
(ZIP)
