# Supplementary figures and images for: Individual Differences in Scotopic Visual Acuity and Contrast Sensitivity: Genetic and Non-Genetic Influences
Source: PLoS One. 2016 Feb 17;11(2):e0148192. doi: 10.1371/journal.pone.0148192 (PMC4757445; doi:10.1371/journal.pone.0148192)

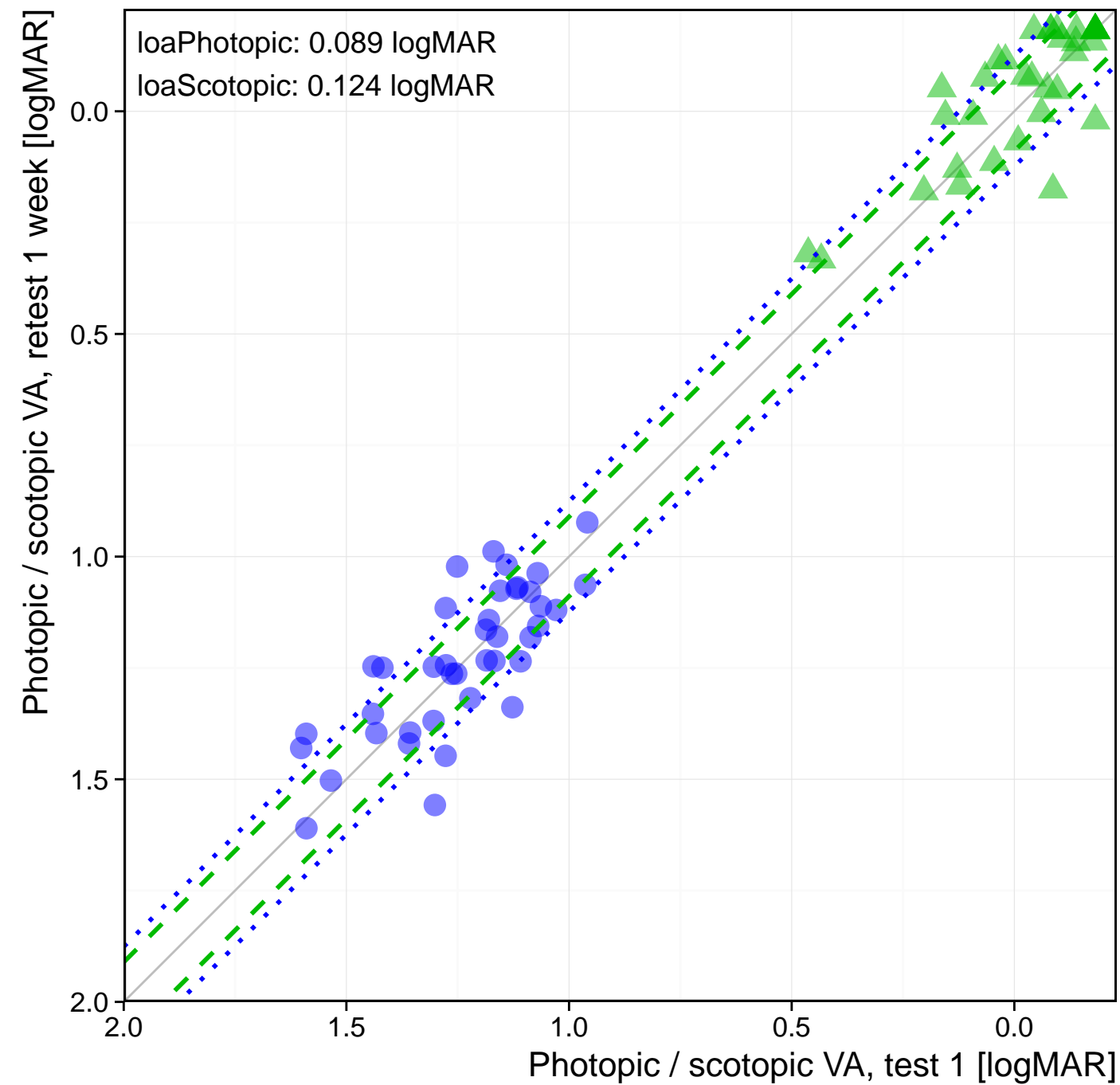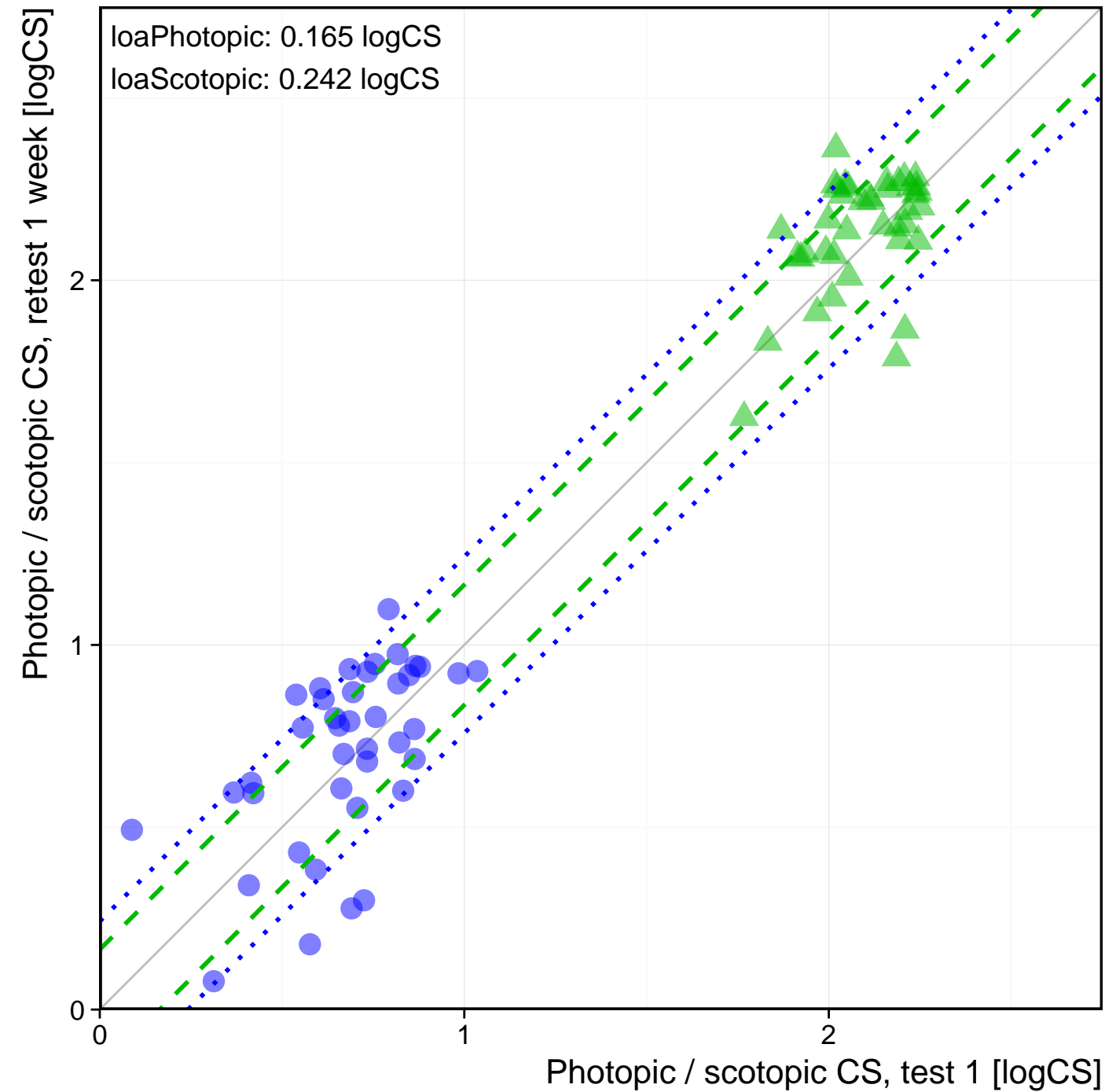

Supplement: S1 Fig — The tests presented here were taken a mean of 87 days apart (SD = 21). Four data sets are depicted: Visual acuity (left panel) and contrast sensitivity (right panel) at photopic luminance (green triangles, near top left and at scotopic luminance (blue discs, near bottom left). Result of the first test on the abscissa, second test on the ordinate. Grey 45°-line is the identity line, next to it the ± limits of agreement (photopic, dashed; scotopic, dotted). Visual acuity in logMAR units have an inverted scale, and contrast sensitivity is in logCSWeber units, meaning that better performance corresponds to the top right for both graphs. (PDF) [file pone.0148192.s001.pdf]

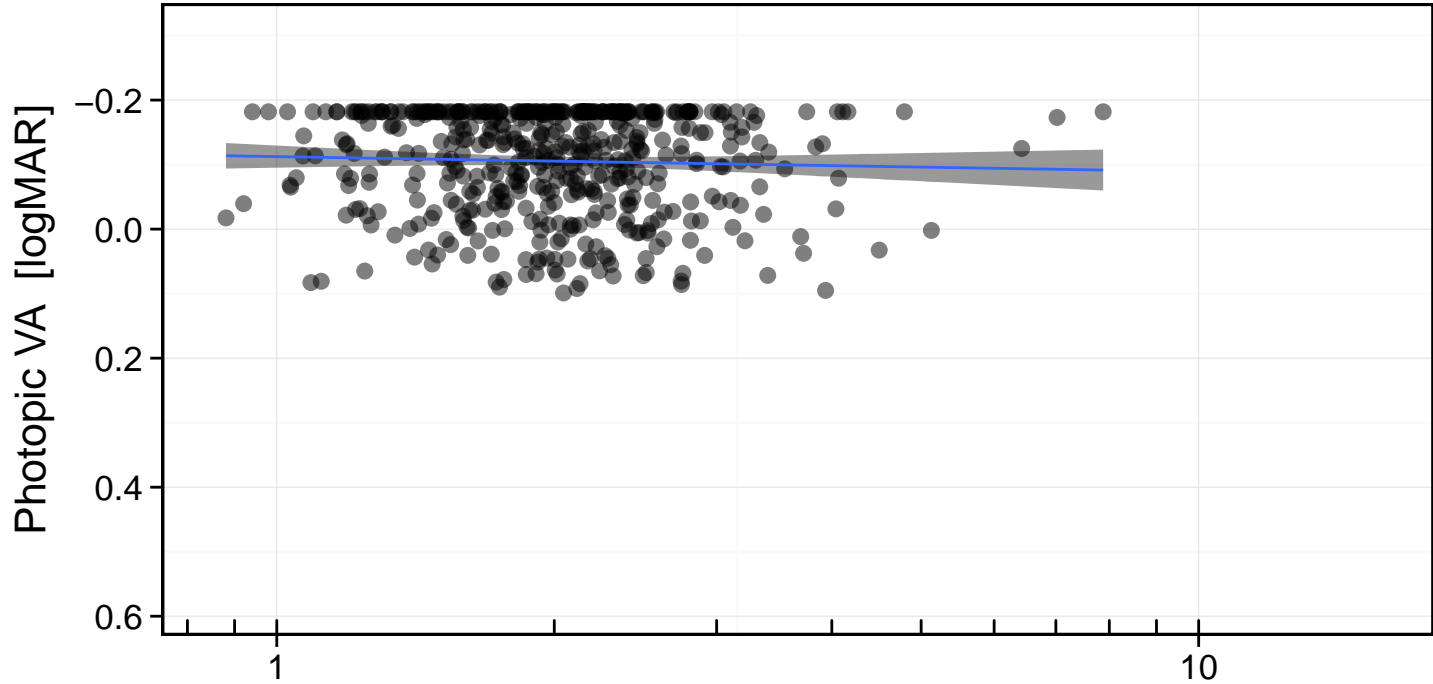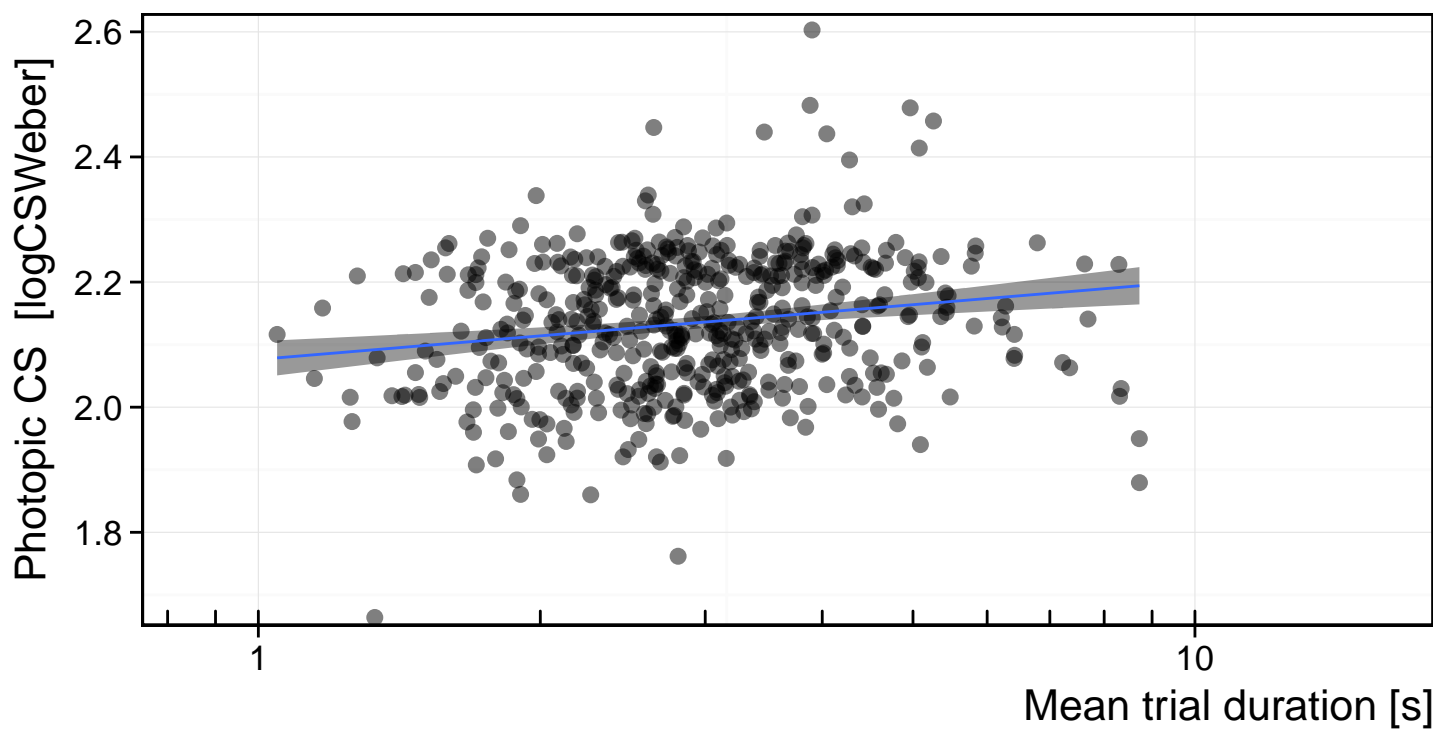

Supplement: S2 Fig — Visual acuity in logMAR units have an inverted scale, and contrast sensitivity is in logCSWeber units, meaning that better performance corresponds to higher scores. Task duration is shown on a log scale and explained <0.1% of the variance in scotopic VA and 3.3% of the variance in scotopic CS. (PDF) [file pone.0148192.s002.pdf]
